# Characterization of lactylation modification subtypes and the promoting role of CCL20 in hepatocellular carcinoma progression

**DOI:** 10.3389/fgene.2025.1605055

**Published:** 2025-07-16

**Authors:** Li-Hong Wu, Liu Yang, Xiang-Xu Wang, Shuang Bai, Xi Yan, Jing Wei, Jun Wang, Fei Tian

**Affiliations:** ^1^ Department of Gastroenterology, Xijing 986 Hospital, Fourth Military Medical University, Xi’an, China; ^2^ Department of Clinical Oncology, Xijing Hospital, Fourth Military Medical University, Xi’an, China; ^3^ Department of Oncology, Xi’an People’s Hospital (Xi’an Fourth Hospital), Xi’an, China

**Keywords:** lactylation modification, hepatocellular carcinoma, prognostic model, CCL20, tumor microenvironment

## Abstract

**Background:**

Hepatocellular carcinoma (HCC) is a highly aggressive and deadly malignancy. Early identification of prognostic risk factors is crucial for guiding clinical management and improving patient outcomes. Lactylation modification plays a pivotal role in tumorigenesis, yet the regulatory mechanisms and prognostic significance of lactylation-related genes in HCC remain insufficiently understood.

**Methods:**

This study screened for prognostically significant lactylation modification-related genes based on their expression levels, integrated with DFS, PFS, and OS data. HCC patients were stratified into three distinct lactylation modification subtypes (C1, C2, C3) using the NMF algorithm. Differentially expressed genes across the three subtypes were identified through an intersection analysis. A lactylation modification-related prognostic model was subsequently constructed using LASSO-Cox and multivariate Cox regression analyses. The CIBERSORT algorithm was utilized to analyze immune cell infiltration. Functional validation of the lactylation-related gene CCL20 in HCC was conducted through *in vitro* experiments using HCC cell lines.

**Results:**

We identified three distinct lactylation modification patterns, with higher lactylation modification levels correlating with worse prognosis in HCC. A six-gene lactylation modification-based prognostic model (LRPS), including FAM83D, ENO1, PFN2, LCAT, PTGR1, and CCL20, was constructed and validated. Overall survival was markedly reduced in the high LRPS group relative to the low LRPS group. The high LRPS group also showed a significantly higher frequency of TP53 mutations. Correlation analysis of immune cell infiltration revealed a significant association between LRPS and the infiltration abundance of M0 macrophages, Tregs, and neutrophils, suggesting that lactylation modification may influence the tumor immune microenvironment. Overexpression of CCL20 in HCC cells significantly enhanced their proliferative and migratory capacities, indicating a key role for CCL20 in HCC progression.

**Conclusion:**

This study established a lactylation modification-based prognostic model that accurately forecasts outcomes in HCC patients. This risk score showed a significant correlation with glucose metabolism and reflected immune cell infiltration patterns. The core model gene, CCL20, promotes HCC cell proliferation and migration, supporting its potential as a valuable prognostic biomarker and a therapeutic target for HCC.

## 1 Introduction

Hepatocellular carcinoma (HCC) ranks as the second contributor to global cancer-related deaths globally, distinguished by its aggressive behavior and unfavorable prognosis (Bray et al., 2024; Singal et al., 2023). While early-stage HCC can be treated surgically, alternative therapies-including ablation, embolization, radiotherapy, targeted therapy, and immunotherapy-are employed for advanced disease (Yang et al., 2024; Psilopatis et al., 2023; Rimassa et al., 2023). Despite continuous advances in treatment strategies, overall survival remains low, primarily because HCC is often diagnosed at an advanced stage (Liu et al., 2016; Yang C. et al., 2023). HCC onset and advancement are shaped by diverse factors, such as genetic mutations, environmental influences, and metabolic shifts (Lin and Li, 2020). Recent studies have increasingly recognized the critical role of metabolic reprogramming in tumor behavior, particularly highlighting lactylation-a novel epigenetic regulatory mechanism-as an important modulator in cancer (Yu et al., 2025).

Lactylation is an emerging post-translational modification that holds the potential to regulate gene expression, cellular functions, and metabolic activity (Zhang et al., 2019; Du et al., 2022). In tumor cells, lactylation plays a pivotal role in immune evasion and tumor progression (Wang et al., 2023; Yang Z. et al., 2023). Although the functional significance of lactylation in cancer is gaining attention, its specific impact on HCC progression and patient prognosis remains unclear. Therefore, a thorough investigation into lactylation-related genes in HCC, integrated with prognostic data, may provide new insights into the clinical management of HCC and uncover novel therapeutic targets.

In this study, we systematically curated a set of lactylation-related genes and constructed a prognostic model for HCC (LRPS) through comprehensive analysis. We employed the Least Absolute Shrinkage and Selection Operator (LASSO) algorithm to eliminate redundant genetic signals and identify key prognostic genes. Additionally, we analyzed the tumor mutation landscape and immune cell infiltration patterns in HCC patients, offering potential insights for immunotherapy. Furthermore, we validated the biological function of the LRPS core gene, CCL20, using *in vitro* experiments with the Hep3B and MHCC97H cell lines. Our findings provide valuable insights into the role of lactylation in regulating HCC progression and patient prognosis, and they highlight potential molecular mechanisms and therapeutic targets for HCC treatment.

## 2 Methods

### 2.1 Data acquisition and lactylation-related gene collection

The mRNA transcriptome data and related clinical details for HCC patients were acquired from The Cancer Genome Atlas (TCGA) database (https://portal.gdc.cancer.gov/), encompassing 370 HCC samples and 50 matched adjacent non-tumor samples. A cohort of 115 HCC samples collected from the GSE76427 dataset (https://www.ncbi.nlm.nih.gov/geo) was served as an independent validation cohort. The mRNA data were converted into Fragments Per Kilobase of transcript per Million mapped reads (FPKM) format and normalized using the “limma” R package. Based on a comprehensive review of the literature, we incorporated 332 previously identified lactylation-related genes (Cheng et al., 2023). A complete list of 332 lactylation-related genes is provided in Supplementary Table S1.

### 2.2 Identification of differentially expressed and prognostic genes

Differentially expressed genes (DEGs) between HCC tumor and adjacent normal tissues were identified using the “limma” R package, with a significance threshold of false discovery rate (FDR) < 0.05 and |log2 fold change (FC)| ≥ 2 (Lu et al., 2022). Univariate Cox regression analysis was conducted to evaluate the prognostic relevance of lactylation-related genes based on recurrence-free survival (RFS), progression-free survival (PFS), and overall survival (OS), with genes exhibiting a P-value <0.05 deemed prognostically significant. The intersection of these gene sets was visualized using a Venn diagram, resulting in the identification of 29 overlapping genes.

### 2.3 Lactylation subtype identification and differential gene expression analysis in HCC

Based on the expression patterns of 29 lactylation-associated genes, HCC patients were grouped into unique lactylation subtypes using the non-negative matrix factorization (NMF) algorithm (An et al., 2025). Expression heatmaps were constructed using the “pheatmap” R package, and survival curves for the identified subtypes were plotted using the “survival” R package. DEGs among the three lactylation subtypes (C1, C2, and C3) were determined using the “limma” package, applying thresholds of FDR <0.05 and |log2 FC| ≥ 2 (Lu et al., 2022). Overlapping DEGs across the three subtypes were identified via a Venn diagram. Furthermore, KEGG pathway enrichment analysis was conducted to elucidate the regulatory pathways connected to these differentially expressed genes (DEGs).

### 2.4 Construction and validation of the lactylation-related prognostic model

The TCGA-LIHC samples were randomly divided into a training set (*n* = 186) and a validation set (*n* = 184). Within the training set, significantly associated lactylation genes were selected using the “glmnet” and “survival” R packages, and a prognostic model was further developed using multivariate Cox regression analysis. LASSO regression analysis was employed to identify potential gene sets for prognostic features (Lin et al., 2024), with gene coefficients for the risk score formula generated using the optimal penalty parameter λ. Patients were stratified into high-risk and low-risk groups based on their risk scores, and survival differences between the two groups were evaluated using the Kaplan-Meier method. To assess the predictive performance of the risk model, receiver operating characteristic (ROC) curves were generated using the “timeROC” R package to evaluate the sensitivity and specificity of the model.

### 2.5 Copy number variation and gene mutation analysis in HCC patients

Copy number variation (CNV) data for the TCGA-LIHC cohort were obtained from the UCSC Xena website (https://xena.ucsc.edu/). Whole-exome sequencing data were downloaded from the TCGA portal (https://portal.gdc.cancer.gov/). Gene mutation analysis was performed using the “maftools” R package, and waterfall plots were generated to illustrate mutation presence and types across different genes.

### 2.6 Immune cell infiltration analysis

To explore the association between lactylation modification-related genes and the HCC immune microenvironment, immune cell infiltration was analyzed using the CIBERSORT algorithm (Newman et al., 2015) across different lactylation subtypes and risk score groups. Leveraging RNA expression data, CIBERSORT employs a deconvolution approach to estimate the infiltration abundance of 22 immune cell subtypes, including T cells, B cells, and macrophages. The relationship between immune cell infiltration, clinical characteristics, and prognostic outcomes was analyzed to further clarify the influence of lactylation modification on the HCC immune microenvironment.

### 2.7 Therapeutic response evaluation

Given the lack of publicly available large-sample hepatocellular carcinoma immunotherapy cohorts, we utilized the IMvigor210 cohort to analyze the immunotherapy response of LRPS. Immunotherapy response data were obtained from the IMvigor210 cohort, comprising patients with urinary tract transitional cell carcinoma treated with the PD-L1 inhibitor atezolizumab. To validate the reliability of the results, we further assessed the difference of LRPS and CCL20 expression between responders and non-responders in the GSE215011 HCC immunotherapy cohort. Responses were categorized as complete response (CR) or partial response (PR) for responders, and stable disease (SD) or progressive disease (PD) for non-responders.

### 2.8 Cell proliferation, migration and invasion assays

Cell proliferation and migration were evaluated using Cell Counting Kit-8 (CCK-8) and wound healing assays, respectively. The proliferation of the HCC cell lines Hep3B and MHCC97H was measured using CCK-8 (Dojindo, Japan) per the manufacturer’s instructions to generate growth curves. For the wound healing assay, a 10 µL pipette tip was used to create a central wound in a six-well plate, followed by culturing in serum-free medium. Cell migration was photographed at 24 h, and the migration rate was calculated as: migration rate = (initial distance - final distance)/initial distance.

Cell migration and invasion were assessed using transwell chambers (8-µm pore size; Corning, United States). For migration assays, cells (5 × 10^4^) in 200 µL of serum-free medium were seeded into the upper chamber. The lower chamber contained 600 µL of complete growth medium supplemented with 10% fetal bovine serum (FBS). For invasion assays, the upper surface of the membrane was pre-coated with Matrigel (Corning, United States). Cells (5 × 10^4^) in 200 µL of serum-free medium were seeded into the upper chamber. The lower chamber contained 600 µL of complete growth medium supplemented with 10% FBS. Chambers were incubated for 24 h at 37°C in a humidified atmosphere of 5% CO_2_. Following incubation, cells adhering to the lower surface of the membrane were fixed and stained with crystal violet. Migrated/invaded cells were quantified using an Olympus microscope.

### 2.9 Statistical analysis

All statistical analyses were performed using R software (version 4.0.3). Differences between groups were assessed using the Mann-Whitney U test or Wilcoxon rank-sum test. Spearman correlation analysis was used to examine the relationship between the lactylation risk prognostic score (LRPS) and immune cell infiltration. Survival outcomes were analyzed via the Kaplan-Meier method, with differences evaluated by the log-rank test. Cox regression analysis assessed the association between lactylation-related genes and HCC prognosis. All tests were two-sided, with P < 0.05 considered statistically significant.

## 3 Results

### 3.1 Multi-omics analysis reveals lactylation gene signatures significantly associated with HCC prognosis

**FIGURE 1:**
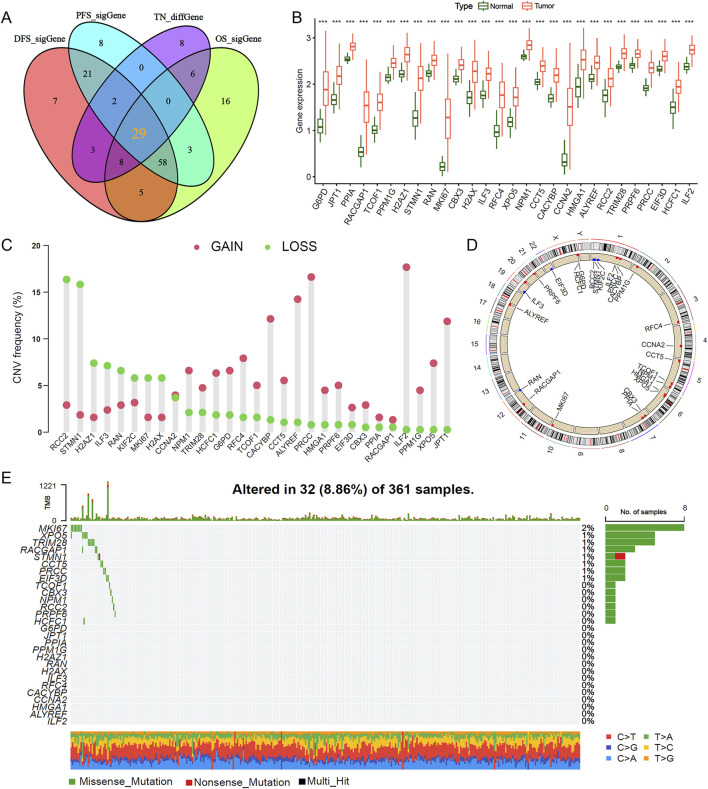
Strategic screening and multi-omics characterization of lactylation-related genes in HCC. **(A,B)** Differential expression analysis of 29 lactylation-related (LR) genes between hepatocellular carcinoma and adjacent normal tissues. **(C)** Copy number variation frequency spectrum of LR genes in the TCGA-LIHC cohort. **(D)** Chromosomal mapping of CNV alterations for the 29 LR genes. **(E)** Mutational landscape depicting frequency and classification of 16 Cu-RGs in TCGA-LIHC patients. ***P < 0.001.

We retrieved mRNA expression data and corresponding clinical information for 370 HCC tissue samples and 50 matched adjacent non-tumor samples from TCGA database. Through a comprehensive analysis of differentially expressed genes and genes associated with HCC prognosis (Supplementary Table S2), we identified 29 lactylation-related genes significantly correlated with HCC prognosis: ALYREF, CBX3, CCNA2, CACYBP, CCT5, CTCF, EIF3D, G6PD, H2AFZ, H2AX, HCF1, ILF2, ILF3, JPT1, MKI67, NPM1, PPM1G, PRPF6, PRRC2, RCC2, RFC4, RAN, STMN1, TCOF1, TRIM28, and XPO5. The overlap between prognostic genes and DEGs was visualized using a Venn diagram (Figure 1A). Subsequent analysis revealed that the expression levels of these 29 DEGs were significantly elevated in HCC tissues compared to adjacent non-tumor tissues (Figure 1B). We further examined copy number variations and somatic mutations in these genes. Notably, RCC2 and STMN1 predominantly exhibited copy number losses, whereas ILF2 and PRRC2 showed copy number gains (Figures 1C,D). Mutation analysis indicated that lactylation-related genes displayed low mutation frequencies, with MKI67 exhibiting the highest mutation rate at 2% (Figure 1E). These findings suggest that lactylation-related genes may influence HCC development and progression primarily through altered gene expression rather than genetic mutations.

### 3.2 Identification of lactylation subtypes in HCC, prognostic analysis, and pathway enrichment

**FIGURE 2:**
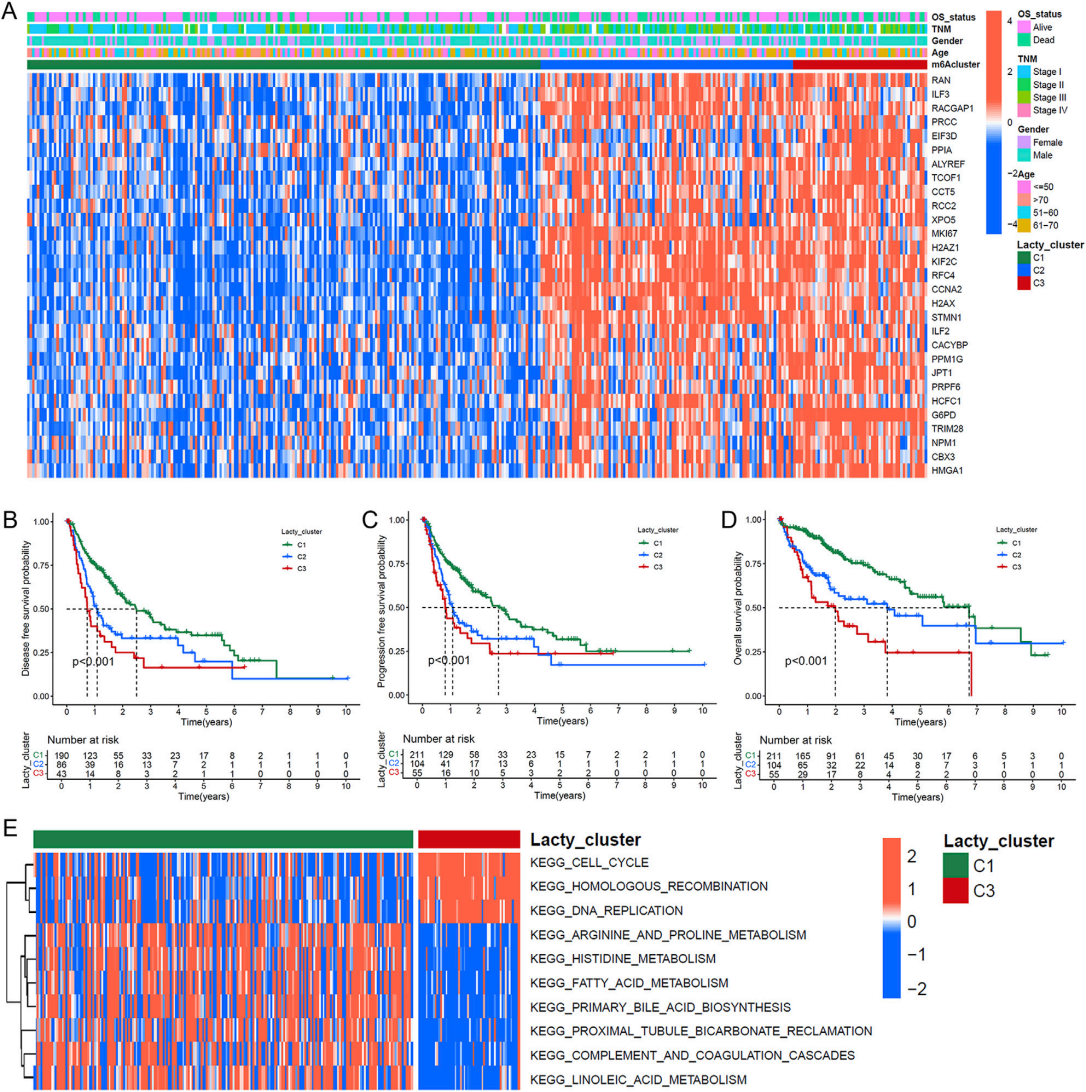
Lactylation-based molecular subtyping and survival impact analysis. **(A)** Expression profile heatmap of 29 LR genes across 370 HCC patients with clinical annotations including lactylation subtypes, survival status, demographic data, and disease staging. **(B–D)** Kaplan-Meier survival analyses comparing disease-free survival **(B)**, progression-free survival **(C)**, and overall survival **(D)** among the three identified lactylation subtypes (C1, C2, and C3). **(E)** GSVA enrichment analysis displaying differential KEGG pathway activation between C1 and C2 lactylation subtypes.

To investigate the role of lactylation modification in HCC, we performed subtype classification using the NMF algorithm based on the expression profiles of lactylation-related genes, identifying three distinct subtypes: C1, C2, and C3 (Supplementary Figure S1). A gene expression heatmap demonstrated that the 29 prognostic lactylation-related genes were most highly expressed in the C3 subtype, followed by C2, with the lowest expression in C1 (Figure 2A). Survival analysis revealed that patients in the C3 subtype had significantly shorter RFS, PFS, and OS compared to those in the C2 and C1 subtypes (Figures 2B–D; Log-rank test, all *P* < 0.05). Pathway enrichment analysis indicated significant enrichment of KEGG pathways related to cell cycle, homologous recombination, and DNA replication in the C3 and C2 subtypes (Figure 2E). In contrast, the C1 subtype was enriched in pathways such as complement and coagulation cascades and arginine and proline metabolism (Supplementary Figure S2A), while the C2 subtype showed enrichment in long-term potentiation pathways (Supplementary Figure S2B). These results suggest that distinct lactylation subtypes in HCC may reflect unique metabolic and regulatory profiles.

### 3.3 Screening of lactylation modification-related genes and construction of a prognostic model

**FIGURE 3:**
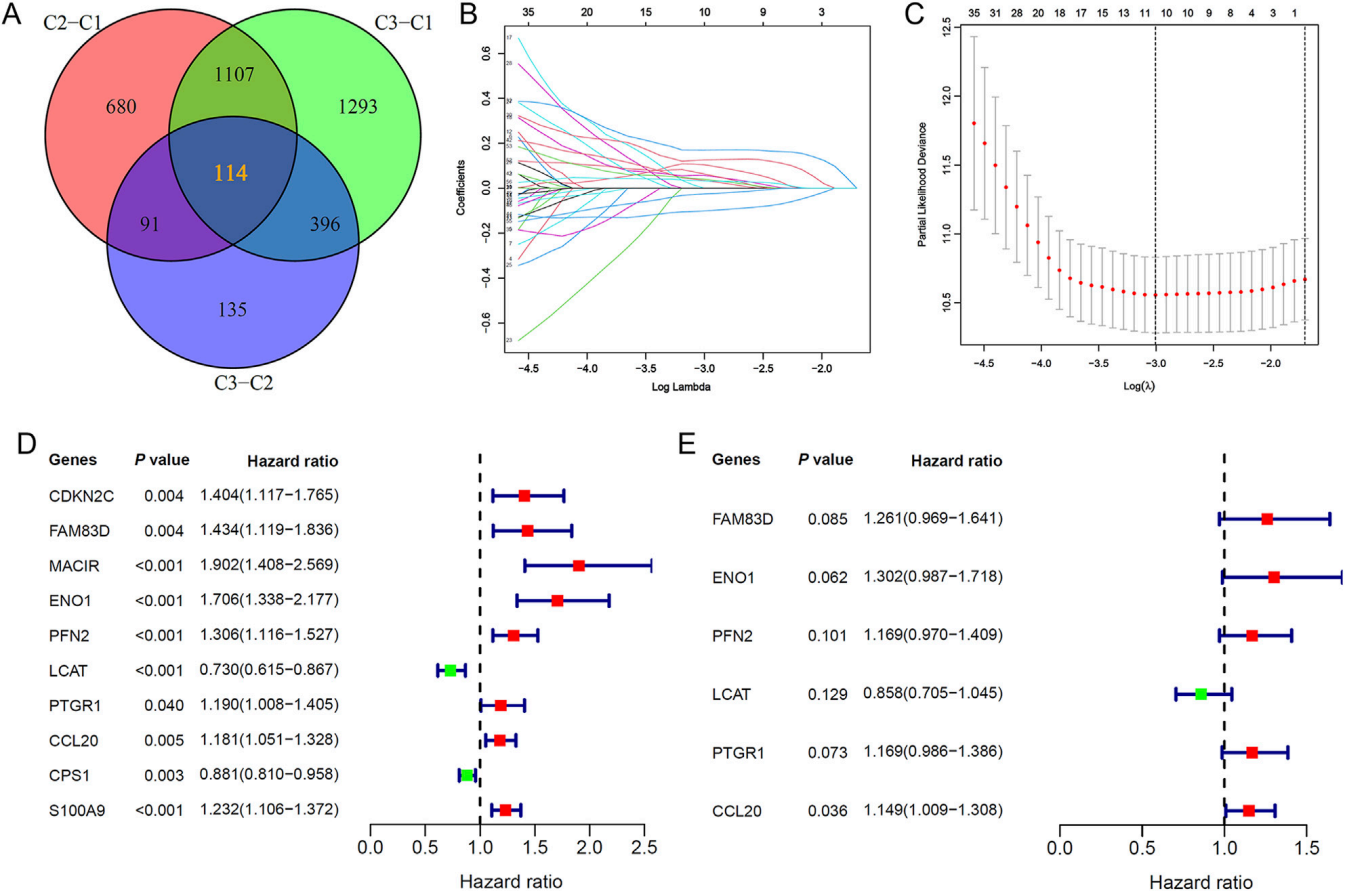
Identification of key lactylation-related genes and prognostic model development. **(A)** Venn diagram highlighting 114 shared lactylation-related genes (LRGs) across the three molecular subtypes. **(B)** Feature selection of 10 LRGs through LASSO regression analysis. **(C)** Ten-fold cross-validation plot validating the LASSO regression model. **(D)** Univariate Cox regression forest plot of the 10 selected LRGs. **(E)** Multivariate Cox regression analysis pinpointed six critical lactylation-related genes (LRGs) for inclusion in the final prognostic signature.

Building on the identified lactylation subtypes, we conducted differential gene expression analysis to identify subtype-associated genes, determining 114 overlapping DEGs via a Venn diagram (Figure 3A). Subsequent LASSO Cox regression analysis, combined with optimal λ value selection, identified 10 lactylation-related genes significantly associated with prognosis (Figures 3B–D). A six-gene prognostic model was then established using multivariate Cox stepwise regression (Figure 3E). The risk score formula for this model, termed Lactylation-Related Prognostic Score (LRPS), is as follows: LRPS = (0.231 × FAM83D_exp) + (0.264 × ENO1_exp) + (0.156 × PFN2_exp) − (0.153 × LCAT_exp) + (0.156 × PTGR1_exp) + (0.139 × CCL20_exp).

### 3.4 Evaluation of the lactylation modification-related prognostic model

**FIGURE 4:**
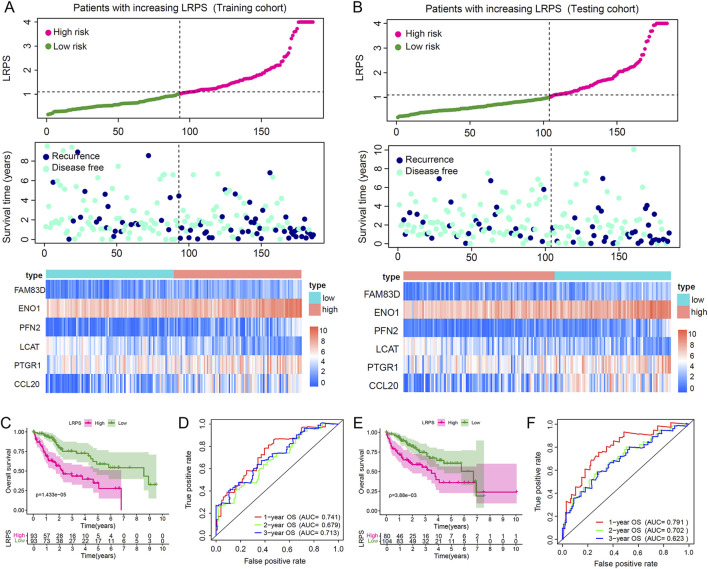
Validation of the lactylation-related prognostic signature (LRPS) in independent cohorts. **(A,B)** Risk score distribution, survival status correlation, and hub gene expression patterns in high versus low LRPS groups across training and testing cohorts. **(C)** Overall survival comparison between high and low LRPS groups in the training cohort. **(D)** Time-dependent ROC curves highlighted the LRPS’s effectiveness in predicting 1-, 2-, and 3-year survival outcomes in the training cohort. **(E)** Overall survival comparison between LRPS groups in the testing cohort. **(F)** Predictive accuracy assessment of LRPS using time-dependent ROC analysis in the testing cohort.

The LRPS enabled the division of HCC patients in both training and validation cohorts into high-risk and low-risk categories. As LRPS increased, patient mortality rates rose significantly, and survival times decreased correspondingly. An expression heatmap revealed that LCAT expression declined with increasing LRPS, whereas the expression of FAM83D, ENO1, PFN2, PTGR1, and CCL20 increased (Figures 4A,B). As expected, Kaplan-Meier survival curves demonstrated significantly lower survival rates in the high LRPS group compared to the low LRPS group (P = 6.915 × 10^−7^; Figures 4C,E). The model’s predictive capability was evaluated via ROC curve analysis, producing 1-year, 2-year, and 3-year OS AUCs of 0.741, 0.679, and 0.713, respectively, in the training cohort (Figure 4D), with identical values in the validation set (Figure 4F). We have similarly validated these findings in an independent HCC cohort from the GSE76427 dataset (Supplementary Figures S3A,B).

### 3.5 Survival analysis of core LRPS genes

**FIGURE 5:**
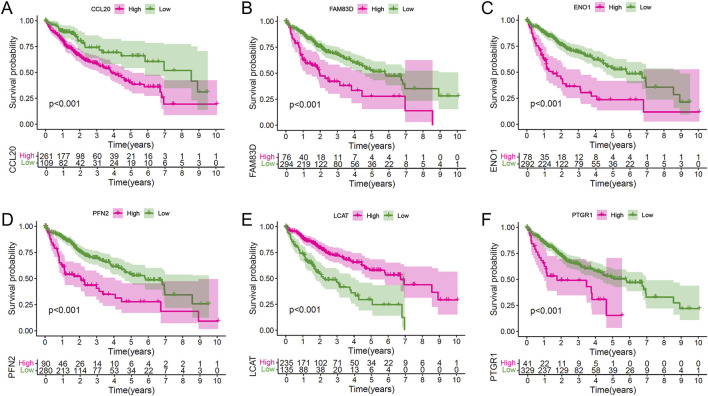
Individual prognostic value of the six hub genes in the combined HCC population. **(A–F)** Kaplan-Meier survival analyses stratifying patients by expression levels of CCL20 **(A)**, FAM38D **(B)**, ENO1 **(C)**, PFN2 **(D)**, LCAT **(E)**, and PTGR1 **(F)**, demonstrating their independent prognostic significance.

To assess the prognostic significance of the core LRPS genes, Kaplan-Meier survival analysis was performed using the entire TCGA dataset. Patients were stratified into high- and low-expression groups based on optimal cut-off values determined by the “surv_cutpoint” function. High expression of FAM83D, ENO1, PFN2, PTGR1, and CCL20 was associated with significantly reduced OS compared to their low-expression counterparts, whereas high LCAT expression correlated with significantly improved OS (Figures 5A–F). Employing the median expression level of the gene in question within the HCC cohort as a threshold, the survival analysis yielded analogous results (Supplementary Figures S4A–F). These findings indicate that LCAT may serve as a protective factor in HCC, while FAM83D, ENO1, PFN2, PTGR1, and CCL20 act as risk factors.

### 3.6 Tumor mutation landscape and clinical feature analysis in high and low LRPS groups

**FIGURE 6:**
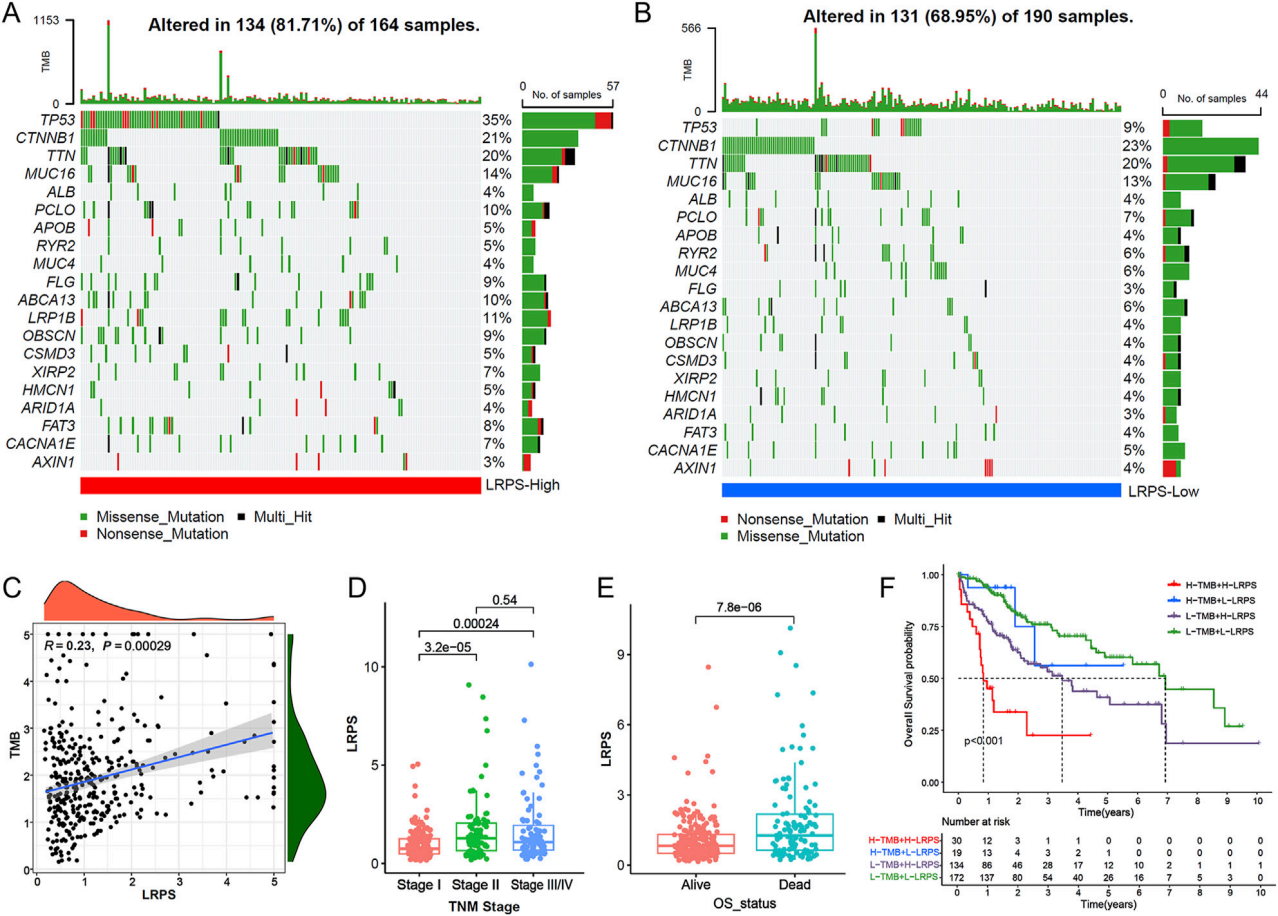
Genomic correlates and clinical relevance of the LRPS. **(A,B)** Mutational landscape visualization comparing somatic mutation patterns between high-LRPS **(A)** and low-LRPS **(B)** groups. **(C)** Association analysis between TMB and LRPS scores. **(D)** LRPS distribution across different TNM stages. **(E)** LRPS comparison between survival outcome groups. **(F)** Integrated survival analysis of patients stratified by both LRPS and TMB status.

We next analyzed the mutation landscape in the high and low LRPS groups. The mutation profile revealed a significantly higher frequency of TP53 mutations in the high LRPS group compared to the low LRPS group, suggesting that TP53 mutations may influence lactylation levels in HCC (Figures 6A,B). Additionally, LRPS exhibited a significant positive correlation with tumor mutation burden (TMB) (Figure 6C; Spearman test, R = 0.23, *P* < 0.001). Clinical feature analysis showed that patients with TNM stages II-IV had significantly higher LRPS scores than those with stage I (Figure 6D; all *P* < 0.001), and deceased patients displayed higher LRPS scores compared to survivors (Figure 6E; *P* < 0.001). Combining TMB and LRPS for prognostic stratification, survival analysis indicated that patients with high LRPS and low TMB exhibited the poorest prognosis, while those with low LRPS and high TMB had the best prognosis (Figure 6F).

### 3.7 Tumor immune microenvironment analysis and therapeutic response evaluation

**FIGURE 7:**
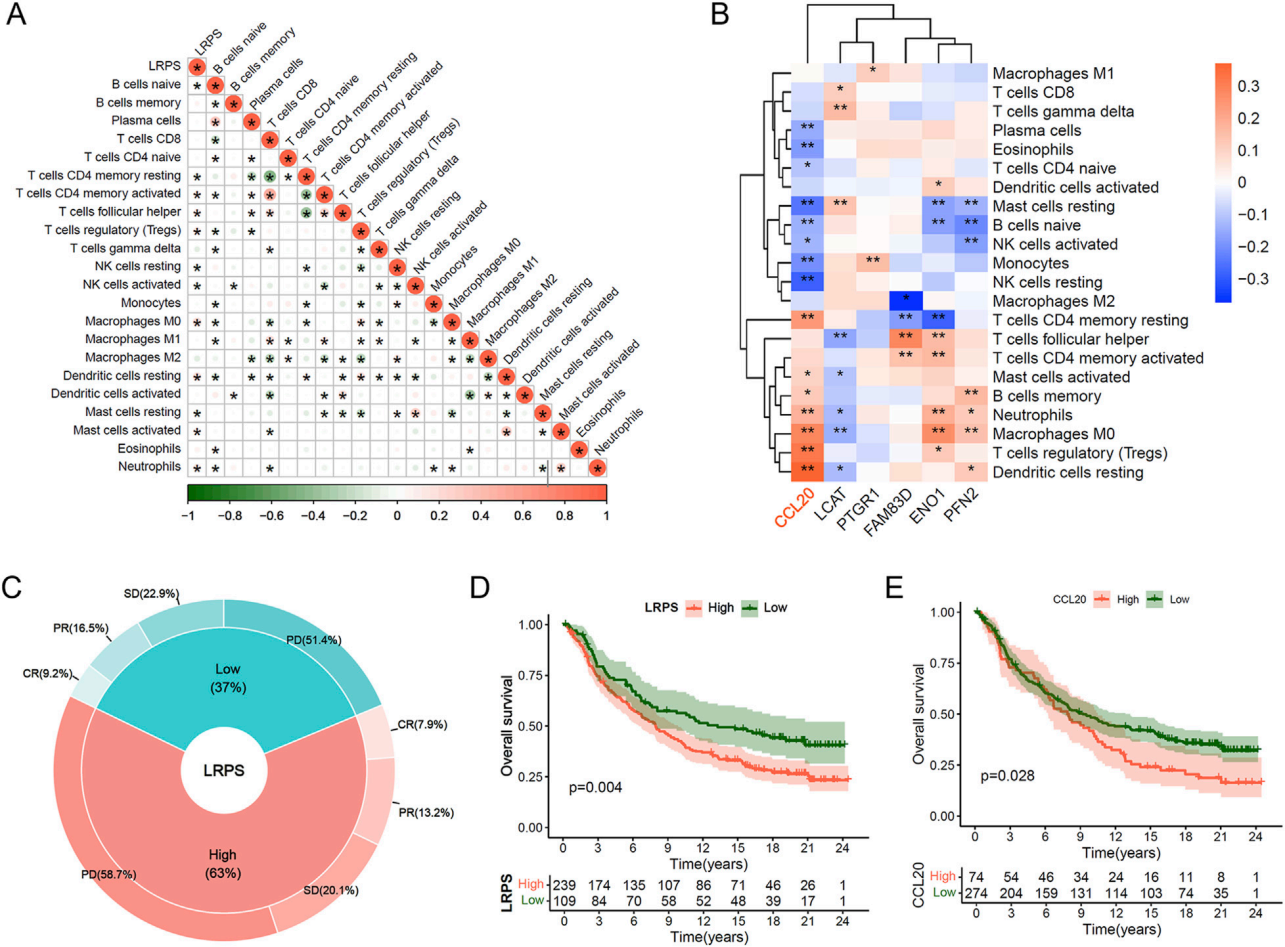
Immunotherapeutic implications of the LRPS. **(A)** Correlation network between LRPS scores and 22 immune cell infiltration profiles. **(B)** Heatmap illustrating relationships between the 6 hub genes and immune cell populations. **(C)** Response rate comparison (CR, PR, SD, and PD) between high and low LRPS groups in the IMvigor-210 immunotherapy cohort. **(D)** Survival analysis comparing LRPS-stratified groups in the IMvigor-210 cohort. **(E)** Survival outcome comparison based on CCL20 expression levels in immunotherapy-treated patients.

Given the critical role of immunotherapy in HCC management, we explored the potential of LRPS in predicting immunotherapy response. Correlation analysis between LRPS and 22 immune cell types revealed significant positive associations with Tregs, M0 macrophages, and neutrophils, and negative associations with NK cells, CD4^+^ memory T cells, and mast cells within the HCC microenvironment (Figure 7A). Further analysis of the six core LRPS genes showed that CCL20 was also significantly positively correlated with Treg, M0 macrophage, and neutrophil infiltration (Figure 7B), suggesting a role for CCL20 in modulating immunosuppressive cell infiltration and tumor progression.

Using data from the IMvigor210 immunotherapy cohort, we evaluated immunotherapy efficacy in high and low LRPS groups (threshold determined by “surv_point”). The high LRPS group exhibited a higher proportion of PD patients (58.7% vs. 51.4%), while CR, PR, and SD proportions were lower compared to the low LRPS group (CR: 7.9% vs. 9.2%; PR: 13.2% vs. 16.5%; SD: 20.1% vs. 22.9%; Figure 7C). Kaplan-Meier survival analysis demonstrated that the high LRPS group experienced significantly diminished OS compared to the low LRPS group (Figure 7D; Log-rank test, *P* = 0.004). Similarly, high CCL20 expression was associated with significantly reduced OS compared to low expression (Figure 7E; Log-rank test, *P* = 0.028). We further validated these findings in HCC immunotherapy cohort (GSE215011). Notably, non-responders exhibited significantly higher LRPS expression compared with responders (Supplementary Figure S5A; Wilcoxon test, *P* = 0.015), while CCL20 showed a marginally significant upregulation trend in non-responders (Supplementary Figure S5B; Wilcoxon test, *P* = 0.056).

### 3.8 CCL20 promotes HCC cell proliferation, migration, invasion, and lactate production *in vitro*

**FIGURE 8:**
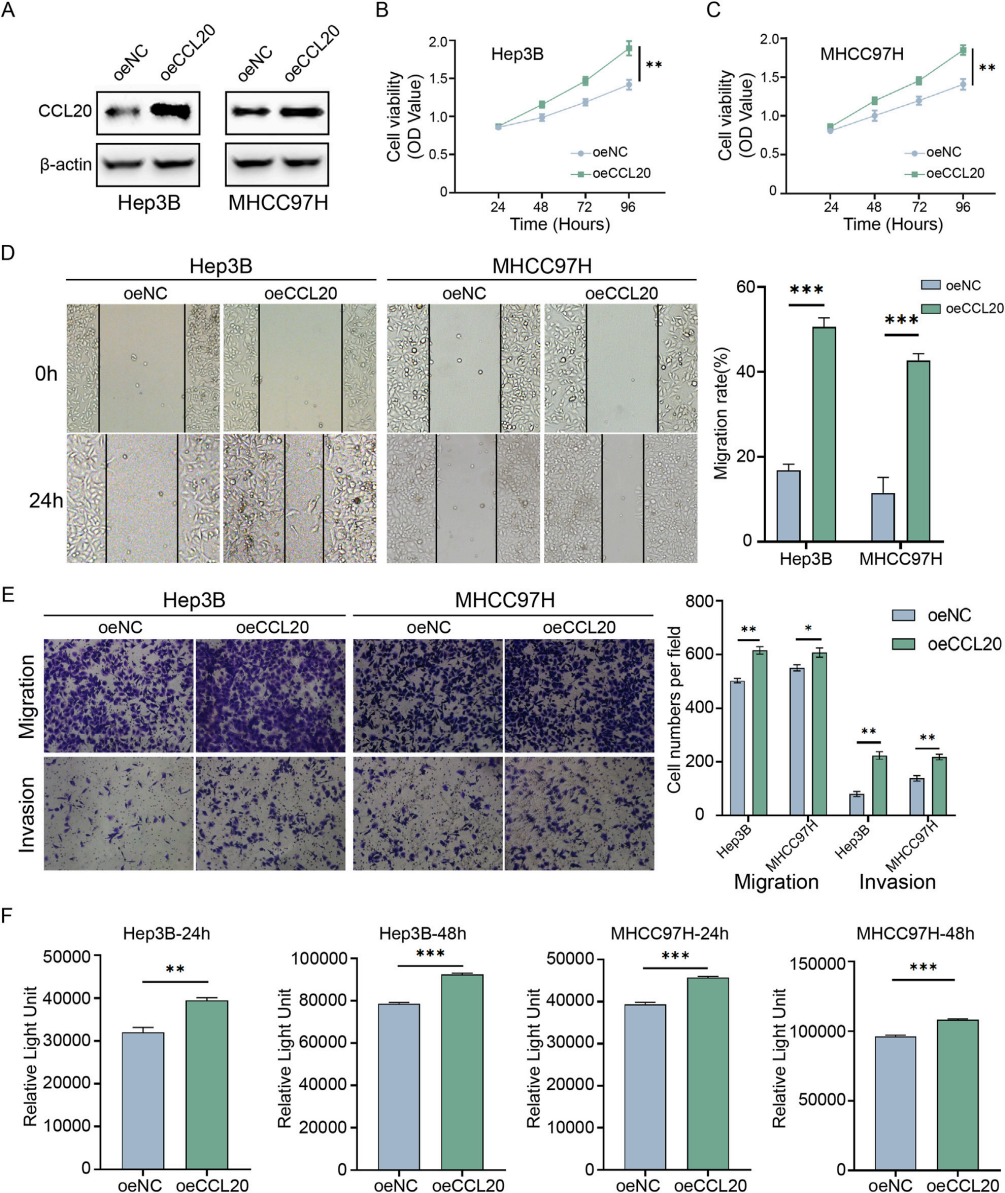
Functional validation of CCL20 in hepatocellular carcinoma cells *in vitro*. **(A)** Western blot analysis showing CCL20 protein expression levels in Hep3B and MHCC97H cells transfected with negative control (NC) or CCL20 overexpression constructs. **(B,C)** Cell viability, assessed by CCK-8 assay, in Hep3B **(B)** and MHCC97H **(C)** cells transfected with NC or CCL20 overexpression constructs at 0, 24, 48, 72, and, 96 h post-transfection. Data are presented as mean ± SD from three independent experiments. **(D)** Wound healing assays demonstrating the migratory capacity of Hep3B and MHCC97H cells overexpressing CCL20 compared to NC controls. Representative images were captured at 0 h and 24 h post-wounding. The right panel shows quantitative analysis of wound closure rates. **(E)** Transwell migration and invasion assays evaluating the migratory and invasive capabilities of Hep3B and MHCC97H cells overexpressing CCL20 compared to NC controls. Representative images and quantitative analysis are shown. **(F)** Lactate levels in the culture medium of Hep3B and MHCC97H cells transfected with NC or CCL20 overexpression constructs. Cells were cultured in lactate-free medium for the indicated times, and lactate concentrations were determined using the Lactate-Glo Assay. Data in **(D–F)** are presented as mean ± SD from three independent experiments. Statistical significance was determined using Student’s t-test. **P < 0.01, ***P < 0.001.

To elucidate the biological functions of CCL20 in HCC, we conducted a series of *in vitro* experiments using Hep3B and MHCC97H cell lines. First, we successfully established CCL20-overexpressing HCC cell models. Western blot analysis confirmed a significant upregulation of CCL20 protein levels in both Hep3B and MHCC97H cells transfected with CCL20 overexpression constructs compared to the negative control (NC) group (Figure 8A). Next, we assessed the effect of CCL20 on cell proliferation using the CCK-8 assay. As shown in Figures 8B,C, overexpression of CCL20 markedly enhanced the viability of both Hep3B and MHCC97H cells at 96 h post-transfection compared to their respective NC groups.

The impact of CCL20 on cell migration was evaluated using wound healing assays. Overexpression of CCL20 significantly accelerated wound closure in both Hep3B and MHCC97H cells at 24 h post-wounding compared to the NC groups (Figure 8D). Quantitative analysis confirmed a significantly higher migratory capacity in CCL20-overexpressing cells. Furthermore, Transwell assays were employed to examine the effects of CCL20 on both cell migration and invasion. The results demonstrated that CCL20 overexpression substantially increased the number of migrated and invaded Hep3B and MHCC97H cells through the Transwell membrane compared to the control cells (Figure 8E). Quantitative analysis revealed a significant enhancement in both migratory and invasive capabilities upon CCL20 overexpression. Given the established link between CCL20 and lactylation, we also investigated whether CCL20 influences lactate production in HCC cells. As depicted in Figure 8F, the Lactate-Glo Assay revealed that overexpression of CCL20 led to a significant increase in lactate levels in the culture medium of both Hep3B and MHCC97H cell.

## 4 Discussion

HCC stands as a prominent driver of cancer-related mortality worldwide, presenting significant therapeutic challenges due to its intricate molecular mechanisms and multifactorial pathogenesis (Yu and Ma, 2024; Yuan et al., 2025). Recent research has underscored the pivotal roles of post-translational modifications in HCC initiation and progression (Lao et al., 2024; Lao et al., 2025; Zhang et al., 2023). Lactylation, a newly identified post-translational modification, has emerged as a critical regulator in various cancers (Lv et al., 2025; He et al., 2024; Gao et al., 2024; Li et al., 2024). However, its specific contributions to HCC and its prognostic implications remain incompletely understood. This study addresses these gaps by constructing a lactylation modification-related prognostic model, offering the first in-depth exploration of its molecular mechanisms and clinical relevance in HCC.

Our initial analysis utilized the NMF algorithm to classify HCC patients into three distinct lactylation modification subtypes (C1, C2, C3) based on the expression profiles of lactylation-related genes. Notably, patients with elevated lactylation levels (C3 subtype) exhibited significantly poorer prognosis, highlighting the substantial influence of lactylation in HCC. This finding aligns with prior studies demonstrating lactylation’s role in tumor cell metabolic reprogramming, proliferation, and invasion. Consequently, lactylation may represent a novel molecular mechanism driving HCC development and progression, providing a foundation for subtype-specific therapeutic strategies. Further analysis led to the development of the Lactylation-Related Prognostic Score (LRPS) model, incorporating six lactylation-related genes: FAM83D, ENO1, PFN2, LCAT, PTGR1, and CCL20. Clinical data revealed that patients in the high LRPS group experienced significantly shorter OS compared to the low LRPS group, accompanied by a markedly higher TP53 mutation frequency. This suggests a close association between lactylation-related genes and HCC prognosis, potentially implicating them in molecular alterations underlying HCC. As a well-established tumor suppressor, TP53 plays a critical role across multiple cancers. The increased TP53 mutation rate in the high LRPS group may reflect lactylation’s regulatory impact on tumor cell metabolism and growth, warranting further mechanistic investigation.

Immune cell infiltration constitutes a vital component of the tumor immune microenvironment (TME), with immune evasion serving as a key determinant of HCC prognosis and treatment response (Pinter et al., 2021; Guo et al., 2022; Wong and Wong, 2025). Our study found significant correlations between LRPS and the infiltration abundance of M0 macrophages, Tregs, and neutrophils, suggesting that lactylation modification may modulate the TME to facilitate immune escape. By influencing immune cell functionality or altering the TME’s composition, lactylation could promote tumor growth and metastasis. These observations position lactylation as a potential target for HCC immunotherapy, offering a novel avenue to counteract immunosuppressive mechanisms.

Our cellular experiments validated the biological significance of CCL20, a lactylation-related gene, in HCC. Overexpression of CCL20 in HCC cells markedly enhanced proliferation and invasion, consistent with previous reports of CCL20’s involvement in tumor cell migration and invasiveness. As a chemokine, CCL20 modulates the TME by recruiting immune cells, potentially fostering tumor cell proliferation and invasion to accelerate HCC progression. Thus, CCL20 not only plays a pivotal role in HCC biology but also emerges as a promising candidate for prognostic assessment and therapeutic targeting.

Despite these insights, our study has limitations. First, while the prognostic model was derived from multi-omics HCC data, the inherent heterogeneity of these datasets may limit its generalizability and precision. Second, although we confirmed CCL20’s functionality in cellular assays, validation in animal models is necessary to substantiate its role *in vivo*. Future studies should elucidate the precise mechanisms by which lactylation influences the HCC immune microenvironment and evaluate the therapeutic potential of targeting lactylation-related genes, particularly CCL20, in preclinical and clinical settings.

## 5 Conclusion

This study integrated whole-exome, transcriptome, and clinical multi-omics data to construct a lactylation modification-based prognostic model for HCC. We elucidated the potential impacts of lactylation on the immune microenvironment, metabolic reprogramming, and tumor progression. Furthermore, preliminary cellular validation of lactylation-related genes, such as CCL20, offers new strategies and targets for personalized HCC therapy. These findings enhance our understanding of lactylation’s role in HCC and pave the way for innovative therapeutic approaches tailored to patient-specific molecular profiles.

## Data Availability

The datasets presented in this study can be found in online repositories. The names of the repository/repositories and accession number(s) can be found in the article/Supplementary Material.
