# Supplementary material for: Characterization of lactylation modification subtypes and the promoting role of CCL20 in hepatocellular carcinoma progression
Source: Front Genet. 2025 Jul 16;16:1605055. doi: 10.3389/fgene.2025.1605055 (PMC12307209; doi:10.3389/fgene.2025.1605055)
Supplement: Supplementary file 2 [file DataSheet1.pdf]

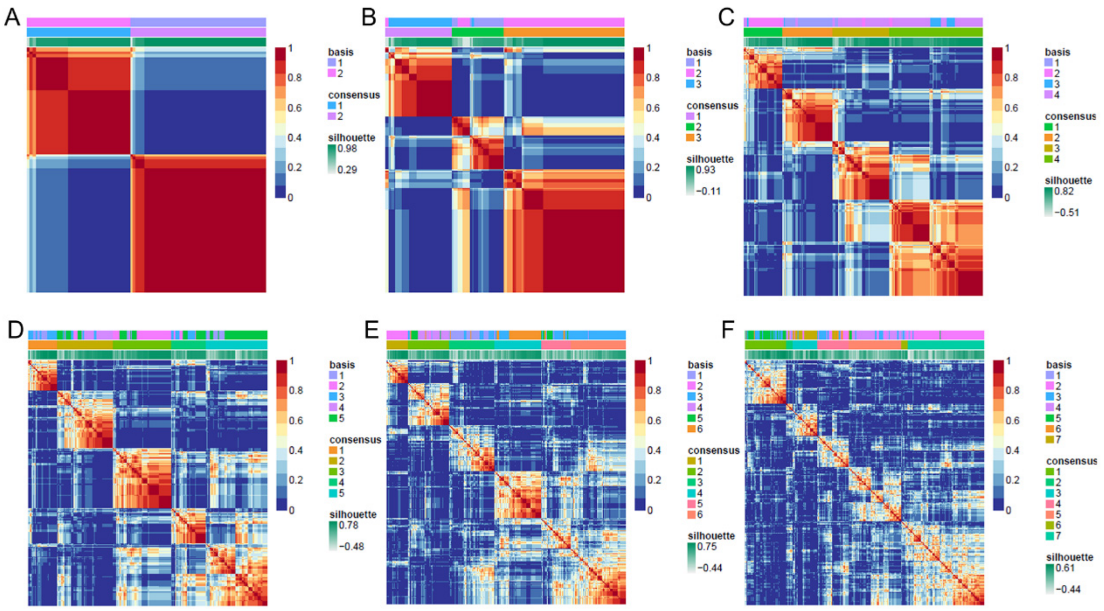

Figure S1. Optimization of Lactylation Subtype Classification Using Non-negative Matrix Factorization. (A-F) Consensus clustering matrices derived from NMF analysis with cluster numbers ranging from 2 to 7, demonstrating the stability and coherence of identified lactylation subtypes at each level of stratification.

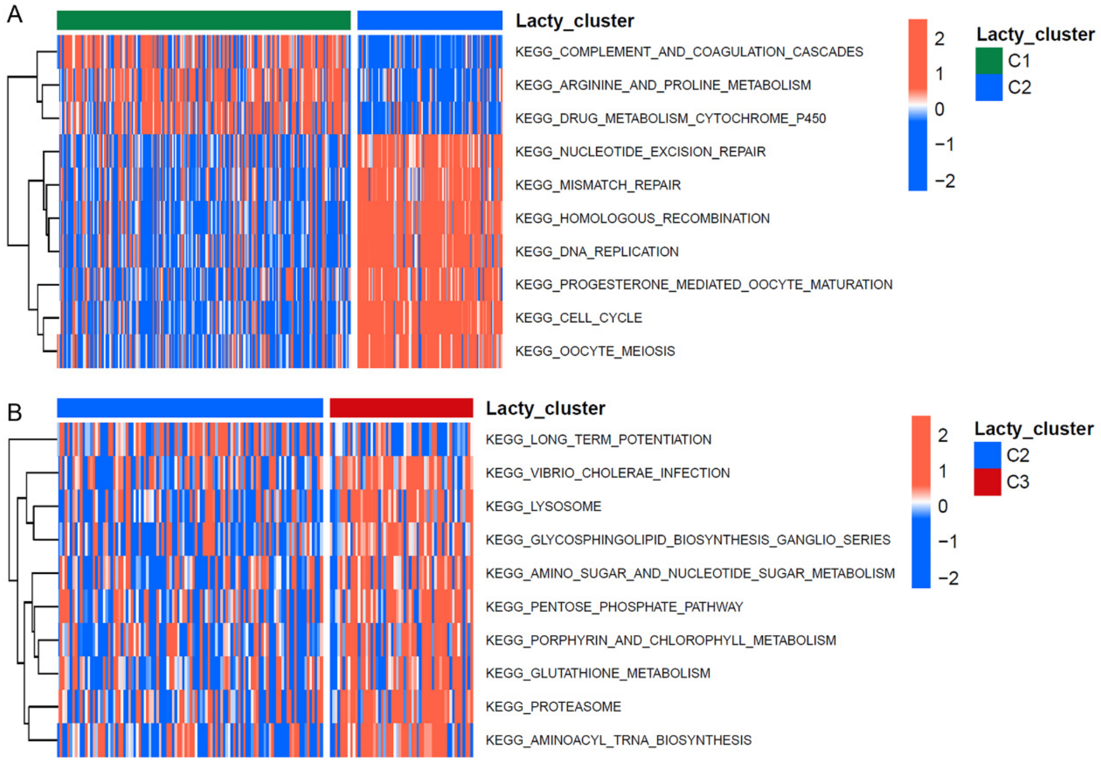

Figure S2. Pathway Enrichment Analysis Revealing Functional Distinctions Between Lactylation Subtypes. (A-B) Differential pathway activation heatmaps displaying significantly enriched KEGG pathways (curated from MSigDB) between lactylation clusters C1 versus C2 (A) and C2 versus C3 (B), highlighting the biological mechanisms underlying subtype-specific characteristics.

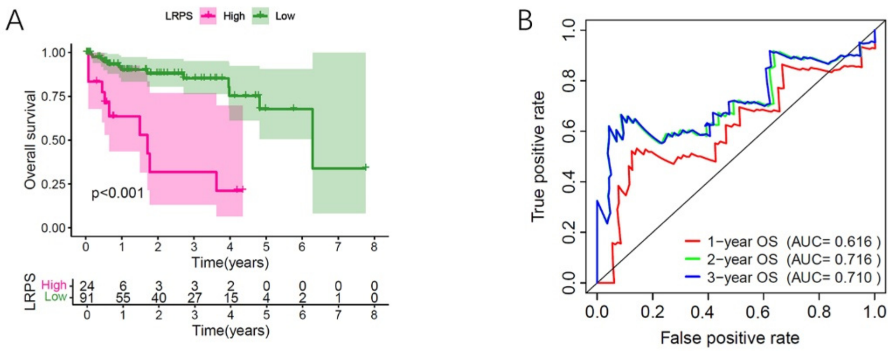

Figure S3. Validation of the Lactylation-related Prognostic Signature (LRPS) in GSE76427 Independent Cohorts. (A) Overall survival comparison between LRPS groups in the GSE76427 independent cohort. (B) Predictive accuracy assessment of LRPS using time-dependent ROC analysis in the GSE76427 independent cohort.

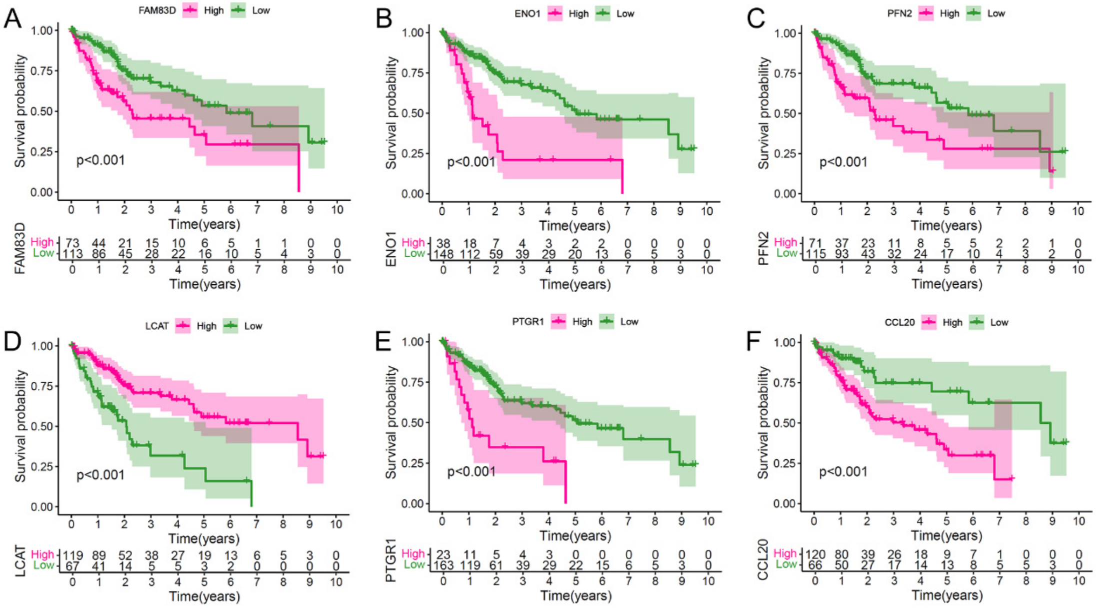

Figure S4. Survival Impact Analysis of 6 Individual Hub Genes in the LRPS Model. (A-F) Kaplan-Meier overall survival analyses in the HCC training cohort stratified by expression levels of FAM83D (A), ENO1 (B), PFN2 (C), LCAT (D), PTGR1 (E), and CCL20 (F), demonstrating the independent prognostic value of each component gene within the lactylation-related prognostic signature.

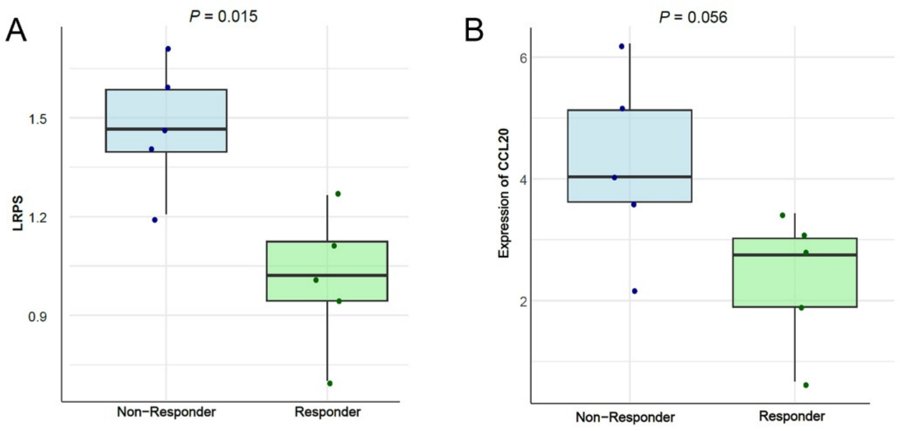

Figure S5. Immunotherapeutic Implications of the LRPS and CCL20 in HCC immunotherapy cohort. (A) Comparison of LRPS between responder and non-responder subgroups in GSE215011 immunotherapy cohort. (B) Comparison of CCL20 expression between responder and non-responder subgroups in GSE215011 immunotherapy cohort.
